# Supplementary material for: Resolving the full spectrum of human genome variation using Linked-Reads
Source: Genome Res. 2019 Apr;29(4):635–45. doi: 10.1101/gr.234443.118 (PMC6442396; doi:10.1101/gr.234443.118)
Supplement: Supplemental Material [file supp_gr.234443.118_Supplemental_longranger-2.2.zip › longranger-2.2.tar.bz2!/lr2.2-public/lib/stan/cmdstan/stan/doc/api/html/functions_s.html]

Stan: Class Members


|  |  |
| --- | --- |
|  | Stan  2.5.0  probability, sampling & optimization |


- Main Page
- Related Pages
- Namespaces
- Classes
- Files

- Class List
- Class Index
- Class Hierarchy
- Class Members

- All
- Functions
- Variables
- Typedefs
- Enumerator
- Related Functions

- \_
- a
- b
- c
- d
- e
- f
- g
- h
- i
- j
- k
- l
- m
- n
- o
- p
- q
- r
- s
- t
- u
- v
- w
- x
- y
- z
- ~


All Classes Namespaces Files Functions Variables Typedefs Enumerations Enumerator Friends Macros Pages

Here is a list of all class members with links to the classes they belong to:

### - s -

- s\_bar\_
  : stan::mcmc::stepsize\_adaptation
- sample()
  : stan::gm::sample
  , stan::mcmc::sample
- sample\_covariance()
  : stan::prob::welford\_covar\_estimator
- sample\_mean()
  : stan::prob::welford\_covar\_estimator
  , stan::prob::welford\_var\_estimator
- sample\_p()
  : stan::mcmc::base\_hamiltonian< M, P, BaseRNG >
  , stan::mcmc::dense\_e\_metric< M, BaseRNG >
  , stan::mcmc::diag\_e\_metric< M, BaseRNG >
  , stan::mcmc::unit\_e\_metric< M, BaseRNG >
- sample\_r
  : stan::gm::statement\_grammar< Iterator >
- sample\_stepsize()
  : stan::mcmc::base\_hmc< M, P, H, I, BaseRNG >
- sample\_variance()
  : stan::prob::welford\_var\_estimator
- samples
  : stan::io::stan\_csv
  , stan::mcmc::chains< RNG >
- sampling
  : stan::io::stan\_csv\_timing
- save\_current\_key\_value\_pair()
  : stan::json::json\_data\_handler
- save\_warmup
  : stan::io::stan\_csv\_metadata
- Scalar
  : stan::agrad::var
- scalar()
  : stan::io::reader< T >
- scalar\_constrain()
  : stan::io::reader< T >
- scalar\_lb()
  : stan::io::reader< T >
- scalar\_lb\_constrain()
  : stan::io::reader< T >
- scalar\_lb\_unconstrain()
  : stan::io::writer< T >
- scalar\_lub()
  : stan::io::reader< T >
- scalar\_lub\_constrain()
  : stan::io::reader< T >
- scalar\_lub\_unconstrain()
  : stan::io::writer< T >
- scalar\_pos()
  : stan::io::reader< T >
- scalar\_pos\_constrain()
  : stan::io::reader< T >
- scalar\_pos\_unconstrain()
  : stan::io::writer< T >
- scalar\_t
  : stan::VectorView< T, is\_array, throw\_if\_accessed >
  , stan::VectorView< const T, is\_array, throw\_if\_accessed >
- scalar\_ub()
  : stan::io::reader< T >
- scalar\_ub\_constrain()
  : stan::io::reader< T >
- scalar\_ub\_unconstrain()
  : stan::io::writer< T >
- scalar\_unconstrain()
  : stan::io::writer< T >
- sd()
  : stan::mcmc::chains< RNG >
- search\_direction()
  : stan::optimization::BFGSUpdate\_HInv< Scalar, DimAtCompile >
  , stan::optimization::LBFGSUpdate< Scalar, DimAtCompile >
- seed
  : stan::io::stan\_csv\_metadata
  , stan::mcmc::base\_hmc< M, P, H, I, BaseRNG >
- seq\_view()
  : stan::math::seq\_view< T, S >
  , stan::math::seq\_view< double, std::vector< int > >
  , stan::math::seq\_view< T, Eigen::Matrix< S, 1, Eigen::Dynamic > >
  , stan::math::seq\_view< T, Eigen::Matrix< S, Eigen::Dynamic, 1 > >
  , stan::math::seq\_view< T, Eigen::Matrix< S, Eigen::Dynamic, Eigen::Dynamic > >
  , stan::math::seq\_view< T, std::vector< S > >
  , stan::math::seq\_view< T, std::vector< std::vector< T > > >
  , stan::math::seq\_view< T, std::vector< T > >
- set\_delta()
  : stan::mcmc::stepsize\_adaptation
- set\_gamma()
  : stan::mcmc::stepsize\_adaptation
- set\_history\_size()
  : stan::optimization::LBFGSUpdate< Scalar, DimAtCompile >
- set\_kappa()
  : stan::mcmc::stepsize\_adaptation
- set\_last\_dim()
  : stan::json::json\_data\_handler
- set\_max\_delta()
  : stan::mcmc::base\_nuts< M, P, H, I, BaseRNG >
- set\_max\_depth()
  : stan::mcmc::base\_nuts< M, P, H, I, BaseRNG >
- set\_mu()
  : stan::mcmc::stepsize\_adaptation
- set\_nominal\_stepsize()
  : stan::mcmc::base\_hmc< M, P, H, I, BaseRNG >
  , stan::mcmc::base\_static\_hmc< M, P, H, I, BaseRNG >
- set\_nominal\_stepsize\_and\_L()
  : stan::mcmc::base\_static\_hmc< M, P, H, I, BaseRNG >
- set\_nominal\_stepsize\_and\_T()
  : stan::mcmc::base\_static\_hmc< M, P, H, I, BaseRNG >
- set\_param\_ranges\_visgen()
  : stan::gm::set\_param\_ranges\_visgen
- set\_stepsize\_jitter()
  : stan::mcmc::base\_hmc< M, P, H, I, BaseRNG >
- set\_T()
  : stan::mcmc::base\_static\_hmc< M, P, H, I, BaseRNG >
- set\_t0()
  : stan::mcmc::stepsize\_adaptation
- set\_type()
  : stan::gm::variable
- set\_user\_defined()
  : stan::gm::function\_signatures
- set\_value()
  : stan::gm::singleton\_argument< T >
- set\_warmup()
  : stan::mcmc::chains< RNG >
- set\_window\_params()
  : stan::mcmc::stepsize\_covar\_adapter
  , stan::mcmc::stepsize\_var\_adapter
  , stan::mcmc::windowed\_adaptation
- set\_zero\_adjoint()
  : stan::agrad::chainable
  , stan::agrad::vari
- sign
  : stan::mcmc::nuts\_util
- signaling\_NaN()
  : std::numeric\_limits< stan::agrad::fvar< T > >
  , std::numeric\_limits< stan::agrad::var >
- simplex()
  : stan::io::reader< T >
- simplex\_constrain()
  : stan::io::reader< T >
- simplex\_decl\_r
  : stan::gm::var\_decls\_grammar< Iterator >
- simplex\_unconstrain()
  : stan::io::writer< T >
- simplex\_var\_decl()
  : stan::gm::simplex\_var\_decl
- singleton\_argument()
  : stan::gm::singleton\_argument< T >
- size()
  : stan::agrad::op\_matrix\_vari
  , stan::agrad::op\_vector\_vari
  , stan::math::coupled\_ode\_system< F, double, double >
  , stan::math::coupled\_ode\_system< F, double, stan::agrad::var >
  , stan::math::coupled\_ode\_system< F, stan::agrad::var, double >
  , stan::math::coupled\_ode\_system< F, stan::agrad::var, stan::agrad::var >
  , stan::math::seq\_view< T, S >
  , stan::math::seq\_view< double, std::vector< int > >
  , stan::math::seq\_view< T, Eigen::Matrix< S, 1, Eigen::Dynamic > >
  , stan::math::seq\_view< T, Eigen::Matrix< S, Eigen::Dynamic, 1 > >
  , stan::math::seq\_view< T, Eigen::Matrix< S, Eigen::Dynamic, Eigen::Dynamic > >
  , stan::math::seq\_view< T, std::vector< S > >
  , stan::math::seq\_view< T, std::vector< std::vector< T > > >
  , stan::math::seq\_view< T, std::vector< T > >
- size\_
  : stan::agrad::op\_matrix\_vari
  , stan::agrad::op\_vector\_vari
  , stan::agrad::stored\_gradient\_vari
  , stan::math::coupled\_ode\_system< F, double, double >
  , stan::math::coupled\_ode\_system< F, double, stan::agrad::var >
  , stan::math::coupled\_ode\_system< F, stan::agrad::var, double >
  , stan::math::coupled\_ode\_system< F, stan::agrad::var, stan::agrad::var >
- size\_cont()
  : stan::mcmc::sample
- size\_of()
  : stan::size\_of\_helper< T, is\_vec >
  , stan::size\_of\_helper< T, true >
- size\_type
  : stan::math::LDLT\_factor< stan::agrad::var, R, C >
  , stan::math::LDLT\_factor< T, R, C >
- SOFTWARE
  : stan::gm::error\_codes
- solve()
  : stan::math::LDLT\_factor< stan::agrad::var, R, C >
  , stan::math::LDLT\_factor< T, R, C >
- solveRight()
  : stan::math::LDLT\_factor< T, R, C >
- split\_arg()
  : stan::gm::argument
- split\_potential\_scale\_reduction()
  : stan::mcmc::chains< RNG >
- stack\_alloc()
  : stan::memory::stack\_alloc
- stage\_
  : stan::gm::var\_size\_validating\_visgen
- stan\_csv\_adaptation()
  : stan::io::stan\_csv\_adaptation
- stan\_csv\_metadata()
  : stan::io::stan\_csv\_metadata
- stan\_csv\_reader()
  : stan::io::stan\_csv\_reader
- stan\_csv\_timing()
  : stan::io::stan\_csv\_timing
- stan\_version\_major
  : stan::io::stan\_csv\_metadata
- stan\_version\_minor
  : stan::io::stan\_csv\_metadata
- stan\_version\_patch
  : stan::io::stan\_csv\_metadata
- start\_array()
  : stan::json::json\_data\_handler
  , stan::json::json\_handler
- start\_nested()
  : stan::memory::stack\_alloc
- start\_object()
  : stan::json::json\_data\_handler
  , stan::json::json\_handler
- start\_text()
  : stan::json::json\_data\_handler
  , stan::json::json\_handler
- statement()
  : stan::gm::statement
- statement\_
  : stan::gm::for\_statement
  , stan::gm::program
  , stan::gm::statement
- statement\_2\_g
  : stan::gm::statement\_grammar< Iterator >
- statement\_2\_grammar()
  : stan::gm::statement\_2\_grammar< Iterator >
- statement\_2\_r
  : stan::gm::statement\_2\_grammar< Iterator >
- statement\_g
  : stan::gm::functions\_grammar< Iterator >
  , stan::gm::program\_grammar< Iterator >
  , stan::gm::statement\_2\_grammar< Iterator >
- statement\_grammar()
  : stan::gm::statement\_grammar< Iterator >
- statement\_r
  : stan::gm::statement\_grammar< Iterator >
- statement\_seq\_r
  : stan::gm::statement\_grammar< Iterator >
- statement\_t
  : stan::gm::statement
- statement\_visgen()
  : stan::gm::statement\_visgen
- statements()
  : stan::gm::statements
- statements\_
  : stan::gm::statements
- std\_vector()
  : stan::io::reader< T >
- step()
  : stan::optimization::BFGSMinimizer< FunctorType, QNUpdateType, Scalar, DimAtCompile >
- step\_size
  : stan::io::stan\_csv\_adaptation
- stepsize\_adaptation()
  : stan::mcmc::stepsize\_adaptation
- stepsize\_adaptation\_
  : stan::mcmc::stepsize\_adapter
  , stan::mcmc::stepsize\_covar\_adapter
  , stan::mcmc::stepsize\_var\_adapter
- stepsize\_adapter()
  : stan::mcmc::stepsize\_adapter
- stepsize\_covar\_adapter()
  : stan::mcmc::stepsize\_covar\_adapter
- stepsize\_var\_adapter()
  : stan::mcmc::stepsize\_var\_adapter
- stored\_gradient\_vari()
  : stan::agrad::stored\_gradient\_vari
- string()
  : stan::json::json\_data\_handler
  , stan::json::json\_handler
- subarguments()
  : stan::gm::categorical\_argument
- subject
  : stan::gm::unary\_op
- success()
  : stan::math::LDLT\_factor< stan::agrad::var, R, C >
  , stan::math::LDLT\_factor< T, R, C >
- sum()
  : stan::common::recorder::sum\_values
  , stan::math::accumulator< T >
- sum\_prob
  : stan::mcmc::nuts\_util
- sum\_values()
  : stan::common::recorder::sum\_values
- system\_function\_name\_
  : stan::gm::integrate\_ode


---

[ Stan Home Page ]

*© 2011–2014,
Stan Development Team.*
